# Clinical Outcomes of Nonsurgical Retreatment in Teeth With Persistent Apical Periodontitis: A Systematic Review

**DOI:** 10.7759/cureus.98601

**Published:** 2025-12-06

**Authors:** Ali M Falatah, Ammar A AlQari, Melaf A Alrwiali, Lujain Altalhi, Nadeen M Alanazi, Abdulrahman K Bin Othman, Mohammed A Alzahrani, Sultan S Alduwaysan, Shurog A Alsaif, Mariam A Ali, Alya J Albalawi

**Affiliations:** 1 Endodontics, Speciality Dental Center, King Fahad Hospital, Madinah, SAU; 2 General Dentistry, Umm Al-Qura University, Mecca, SAU; 3 Dentistry, Jouf University, Al-Jouf, SAU; 4 Dentistry, King Saud Bin Abdulaziz University for Health Sciences, Riyadh, SAU; 5 General Dentistry, PEDS Pediatric Dentistry and Orthodontics, Riyadh, SAU; 6 General Dentistry, Al Baha University, Al Baha, SAU; 7 General Dentistry, King Saud University, Riyadh, SAU; 8 General Dentistry, King Saud Bin Abdulaziz University for Health Sciences, Riyadh, SAU; 9 General Dentistry, Cairo University, Giza, BHR; 10 General Dentistry, Al-Rabwa Medical Center, Riyadh, SAU

**Keywords:** endodontics, nonsurgical retreatment, outcome assessment, root canal retreatment, treatment outcome

## Abstract

Persistent apical periodontitis is a common cause of endodontic failure. While nonsurgical retreatment (NS-ReTx) is the typical first-line option used to manage this condition, its reported success varies depending on clinical, radiographic, and methodological factors despite recent advances that improve case management. This systematic review aimed to assess the clinical and radiographic outcomes of NS-ReTx in teeth with persistent apical periodontitis. Following the Preferred Reporting Items for Systematic Reviews and Meta-Analyses (PRISMA) 2020 guidelines, the databases of PubMed/MEDLINE, Embase, Cochrane Central Register of Controlled Trials (CENTRAL), Scopus, Web of Science, and grey literature were searched for randomized and non-randomized clinical studies and case series involving ≥10 teeth reporting outcomes after orthograde retreatment from January 1988 to September 2025. The primary outcome was periapical healing assessed through radiography and/or cone-beam computed tomography (CBCT), and secondary outcomes were tooth survival and prognostic factors. Risk of bias was appraised using the Cochrane Risk of Bias 2.0 (RoB 2) tool, ROBINS-I, or the Newcastle-Ottawa Scale, as appropriate, and findings were synthesized narratively due to heterogeneity in outcome definitions, imaging modalities, and follow-up intervals. Thirty studies, comprising randomized controlled trials (RCTs) and cohorts, met the inclusion criteria. Across these studies, reports using strict radiographic criteria or CBCT generally described lower apparent healing rates than those using looser criteria and 2D radiographs, although the magnitude of this difference could not be reliably quantified across studies. Additionally, a favorable prognosis was consistently associated with the absence of preoperative lesions, smaller lesion sizes, adequate obturation and coronal seals, and specialist operators. Overall, NS-ReTx provides favorable outcomes for persistent apical periodontitis. Future research should standardize outcome definitions using validated frameworks (such as periapical index (PAI)-based radiographic criteria and CBCT-specific scoring systems) and adopt longer, more uniform follow-up periods to enable robust quantitative synthesis.

## Introduction and background

Root canal treatments aim to prevent reinfection of the tooth by cleaning and shaping the root canal system, followed by the application of a three-dimensional seal [1,2]. Although primary root canal therapy is generally predictable with a high degree of success [3-6], failures still occur; recent reports estimate a 14-16% failure rate after initial treatment [3,7]. Post-treatment disease typically presents as persistent apical periodontitis, and three management options are considered in this situation: nonsurgical retreatment (NS-ReTx), surgical endodontics, or extraction. Selecting the best approach involves considering evidence from robust studies that consider the benefit-risk balance [8,9]. Persistent apical periodontitis after root canal treatment is multifactorial in origin [5,7,9], with common contributors including residual intraradicular infection, missed anatomy, inadequate disinfection, under- or over-extension of root fillings, and loss of coronal seal (coronal leakage) [5,9]. Patient-level factors such as systemic health and smoking, along with tooth-level features like lesion size and posterior tooth morphology, further influence healing and long-term function [7]. To treat this condition, NS-ReTx aims to re-establish asepsis by removing existing materials, negotiating missed canals, enhancing irrigation/activation, and achieving a dense three-dimensional obturation [1,2,9]. Compared with surgery or extraction, NS-ReTx preserves tooth structure, maintains proprioception, and avoids surgical morbidity when the canal system can be re-accessed. Earlier systematic reviews report wide variations in favorable outcomes (28-100%) [4-6,10], which is likely due to differences in outcome definitions, follow-up periods, operators, and historical techniques [10]; for example, reviews that included teeth with large preoperative lesions and applied strict radiographic criteria tended to report success rates toward the lower end of this range, whereas those focusing on specialist-treated cases with more favorable preoperative status and looser radiographic criteria reported success rates closer to the upper end. However, recent innovations, such as rotary or reciprocating nickel-titanium (NiTi) systems, advanced irrigation and activation protocols, magnification, and bioceramic sealers, have transformed current clinical practices. In specialist-treated series involving well-restorable teeth and often limited or absent preoperative periapical lesions, reported success rates approach ~94% [11], and these developments warrant an updated evaluation of outcomes. Across the existing evidence base, healing is predominantly assessed using two-dimensional periapical radiographs, and operational definitions of "success" or "healing" vary considerably [4-6,10]. These variations in radiographic criteria and follow-up windows contribute to the wide range in reported outcomes after root canal treatment and retreatment [7-10]. Furthermore, most outcome data is still derived from conventional radiography, even though cone-beam computed tomography (CBCT) imaging offers three-dimensional assessment and greater sensitivity in detecting periapical changes. This could create discrepancies when comparing outcomes. In practice, case selection for NS-ReTx requires balancing lesion size, quality of previous treatment, coronal restoration integrity, operator expertise, and patient preferences. These are all factors that directly impact prognosis and guide patient counselling and consent, as well as treatment sequencing relative to surgery or extraction with implant-supported rehabilitation [8,9].

Despite numerous primary studies and several reviews, uncertainty persists about the true clinical success of NS-ReTx, particularly when outcomes are judged by CBCT instead of periapical radiography and when strict rather than loose healing criteria are applied. The present systematic review, therefore, aims to synthesize the available evidence on the clinical and radiographic outcomes of NS-ReTx for persistent apical periodontitis, with particular focus on outcome definitions, imaging modality, and key prognostic factors relevant to contemporary endodontic practice.

## Review

Methods

Protocol Design and Database Search Strategy 

The review methodology was designed to evaluate the clinical outcomes of nonsurgical endodontic retreatment in teeth with persistent apical periodontitis. This systematic review was conducted in accordance with the Preferred Reporting Items for Systematic Reviews and Meta-Analyses (PRISMA) 2020 guidelines [12].

Inclusion Criteria

The Population, Intervention, Comparison, Outcomes, and Study (PICOS) framework [13] was applied to identify eligible studies. The following inclusion criteria were used:

Population (P): We included human studies involving permanent teeth with persistent apical periodontitis following previous root canal treatment, where the diagnosis was confirmed radiographically and/or clinically. Participants of any age, sex, or geographic location were eligible.

Intervention (I): The intervention of interest was nonsurgical endodontic retreatment, defined as orthograde re-instrumentation, disinfection, and re-obturation of root canals. Studies were eligible whether they used conventional methods or modern techniques such as rotary or reciprocating instruments, advanced irrigation methods, or bioceramic sealers.

Comparison (C): Where available, comparator groups included surgical retreatment, extraction with or without implant placement, or no intervention. Studies without comparators were eligible if they reported defined retreatment outcomes.

Outcomes (O): The primary outcome was healing of apical periodontitis, assessed clinically and/or radiographically. For the purpose of this review, "strict" healing criteria were defined as complete radiographic resolution of the periapical lesion (for example, a normal periapical appearance or low periapical index (PAI) score/complete CBCT bone fill) in an asymptomatic, functional tooth, whereas "loose" criteria allowed for a reduction in lesion size and absence of symptoms despite residual radiolucency. Individual studies were classified as using strict or loose criteria based on their reported outcome definitions. Secondary outcomes included prognostic factors influencing healing, such as lesion size, tooth type, and coronal restoration status.

Study design (S): Randomized controlled trials (RCTs), prospective or retrospective cohort studies, and case-control studies were included. Case series with ≥10 cases and systematic reviews were considered for background context only; they were not included in the formal qualitative synthesis, risk-of-bias assessment, or certainty of evidence (Grading of Recommendations Assessment, Development, and Evaluation (GRADE)) evaluation. To be considered eligible, studies had to have follow-up periods of ≥12 months.

Exclusion Criteria 

Studies that met the following criteria were excluded from analysis.

Study design: Case reports, case series with fewer than 10 cases, narrative reviews, editorials, letters, expert opinions, animal or in vitro studies, conference abstracts, and unpublished theses were excluded.

Population: Studies including only primary root canal treatment cases, patients with systemic conditions directly affecting periapical healing (unless outcomes were reported separately), or teeth with vertical root fractures, resorption, or non-restorable structures were excluded.

Intervention: Studies limited to surgical retreatment as the sole approach, studies that combined surgical and nonsurgical approaches at baseline, and studies with retreatment procedures that were unclear, inadequately described, or not orthograde in nature were excluded.

Outcomes: Studies without clearly defined clinical or radiographic outcomes or those reporting only surrogate markers, such as microbial reduction, were excluded.

Follow-up: Studies with follow-up periods shorter than 12 months were excluded.

Publication: Non-English publications without full translation and literature that are not peer-reviewed were excluded.

Search Strategy

A comprehensive electronic literature search was conducted in accordance with PRISMA 2020 guidelines [12] to identify studies evaluating the clinical outcomes of nonsurgical root canal retreatment with defined clinical and/or radiographic healing criteria. The strategy was developed in consultation with an experienced health sciences librarian and included five major databases (PubMed/MEDLINE, Embase, Scopus, Cochrane Central Register of Controlled Trials (CENTRAL), and Web of Science Core Collection) from January 1988 to September 2025. This broad time frame was chosen to capture long-term outcome studies and to encompass the evolution of endodontic technologies (e.g., NiTi instruments, magnification, and advanced irrigation protocols), which was explicitly considered when interpreting heterogeneity between older and more contemporary cohorts.

In addition to electronic databases, grey literature sources (OpenGrey, ProQuest Dissertations and Theses Global, ClinicalTrials.gov, and World Health Organization (WHO) International Clinical Trials Registry Platform (ICTRP)) were screened to minimize publication bias. Reference lists of included studies and leading endodontic journals were also hand searched. All search results were imported into EndNote X9 (Clarivate Analytics, London, United Kingdom) [14] for reference management, and duplicate records were removed prior to screening.

 The detailed database-specific search strategies are summarized in Table 1.

**Table 1:** Database search strategy NCBI: National Center for Biotechnology Information; RCT: randomized controlled trial; CENTRAL: Cochrane Central Register of Controlled Trials; TS: topic search (Web of Science search field); MeSH: Medical Subject Headings (PubMed indexing terms)

Database	Platform/interface	Coverage years	Exact search terms/queries	Filters applied	Records retrieved	After deduplication	Date of last search
PubMed (MEDLINE)	NCBI	1988-2025	("non-surgical retreatment"[Title/Abstract] OR "orthograde retreatment"[Title/Abstract] OR "endodontic retreatment"[Title/Abstract]) AND ("apical periodontitis"[MeSH] OR "periapical lesion"[Title/Abstract]) AND ("treatment outcome"[MeSH] OR healing[Title/Abstract] OR success[Title/Abstract])	Humans, English, RCT + observational	1012	681	10 Sept 2025
Embase	Ovid	1988-2025	('nonsurgical retreatment':ab,ti OR 'orthograde retreatment':ab,ti OR 'endodontic retreatment':ab,ti) AND ('apical periodontitis'/exp OR 'periapical lesion':ab,ti) AND ('treatment outcome'/exp OR healing:ab,ti OR success:ab,ti)	Humans, English, clinical trials	747	496	10 Sept 2025
CENTRAL	Wiley	Inception to 2025	("endodontic retreatment" OR "nonsurgical retreatment" OR "orthograde retreatment") in Title Abstract Keyword AND ("apical periodontitis" OR "periapical lesion") AND (healing OR outcome OR success)	No filters	195	172	10 Sept 2025
Scopus	Elsevier	1988-2025	TITLE-ABS-KEY("nonsurgical retreatment" OR "orthograde retreatment" OR "endodontic retreatment") AND TITLE-ABS-KEY("apical periodontitis" OR "periapical lesion") AND TITLE-ABS-KEY("outcome" OR "healing" OR "success")	No filters	879	621	10 Sept 2025
Web of Science (Core Collection)	Clarivate	1988-2025	TS=("nonsurgical retreatment" OR "orthograde retreatment" OR "endodontic retreatment") AND TS=("apical periodontitis" OR "periapical lesion") AND TS=("treatment outcome" OR "healing" OR "success")	English only	479	355	10 Sept 2025
Grey literature (OpenGrey, ProQuest Dissertations and Theses Global)	Various	Inception to 2025	("endodontic retreatment" OR "nonsurgical retreatment") AND ("apical periodontitis" OR "periapical disease")	No filters	147	62	10 Sept 2025

Study Selection and Screening

All records retrieved from electronic databases and grey literature were exported into EndNote X9 [14], where duplicate citations were identified and removed. The deduplicated library was then imported into Rayyan Qatar Computing Research Institute (QCRI) (Rayyan Systems Inc., Cambridge, Massachusetts, United States) [15] for blinded, independent screening.

Screening Procedure

Two reviewers independently screened titles and abstracts against the predefined inclusion and exclusion criteria. Studies that clearly did not meet the eligibility criteria were excluded at this stage. Discrepancies were resolved by discussion, and a third reviewer acted as arbiter if consensus could not be reached. Full texts were obtained for potentially eligible records and assessed in detail against the inclusion criteria. Reasons for exclusion at the full-text stage (e.g., wrong intervention, absence of strict healing criteria, lack of clinical outcome data, case reports, in vitro studies) were recorded for transparency.

Inter-rater Reliability

To ensure methodological rigor, agreement between reviewers was assessed using Cohen's kappa (κ) [16] during both title/abstract and full-text screening. Agreement was interpreted using Landis and Koch's benchmarks [17], with κ>0.75 regarded as excellent.

Data Collection Process

Two reviewers independently extracted data using a previously tested Microsoft Excel form (Microsoft Corporation, Redmond, Washington, United States) [18]. Disagreements were resolved by consensus or by a third reviewer's input. When outcome data were incomplete or unclear, study authors were contacted by email; if they did not respond within four weeks, available data were retained and the limitation documented.

Data Items

We extracted the following data items: (1) study details, including first author, country, year, design, sample size, and follow-up duration; (2) participant and tooth characteristics, such as number of patients and teeth, age and sex where reported, tooth type and location, and preoperative signs and symptoms; (3) treatment details, including quality of previous root filling, retreatment instrumentation/irrigation/obturation, use of magnification, sealer type, and operator category; (4) outcomes, comprising primary outcomes of periapical healing under strict radiographic and clinical criteria and secondary outcomes of functional tooth retention, symptom resolution, survival, adverse events, and reported prognostic factors; and (5) methodological data points required for risk-of-bias assessment.

Risk-of-Bias Assessment

Two reviewers independently assessed the risk of bias using standardized tools appropriate for the study design. RCTs were evaluated using the Cochrane Risk of Bias 2.0 (RoB 2) tool, which examines five domains: (1) bias in the randomization process, (2) deviations from intended interventions, (3) missing outcome data, (4) measurement of outcomes, and (5) selection of reported results [19]. Cohort and case-control studies were assessed using the Newcastle-Ottawa Scale (NOS), which evaluates selection, comparability, and outcome domains [20]. Disagreements between reviewers were resolved through discussion or arbitration by a third reviewer. Certainty of evidence for the main outcomes was assessed using the GRADE approach [21].

Results

Study Selection

**Figure 1:**
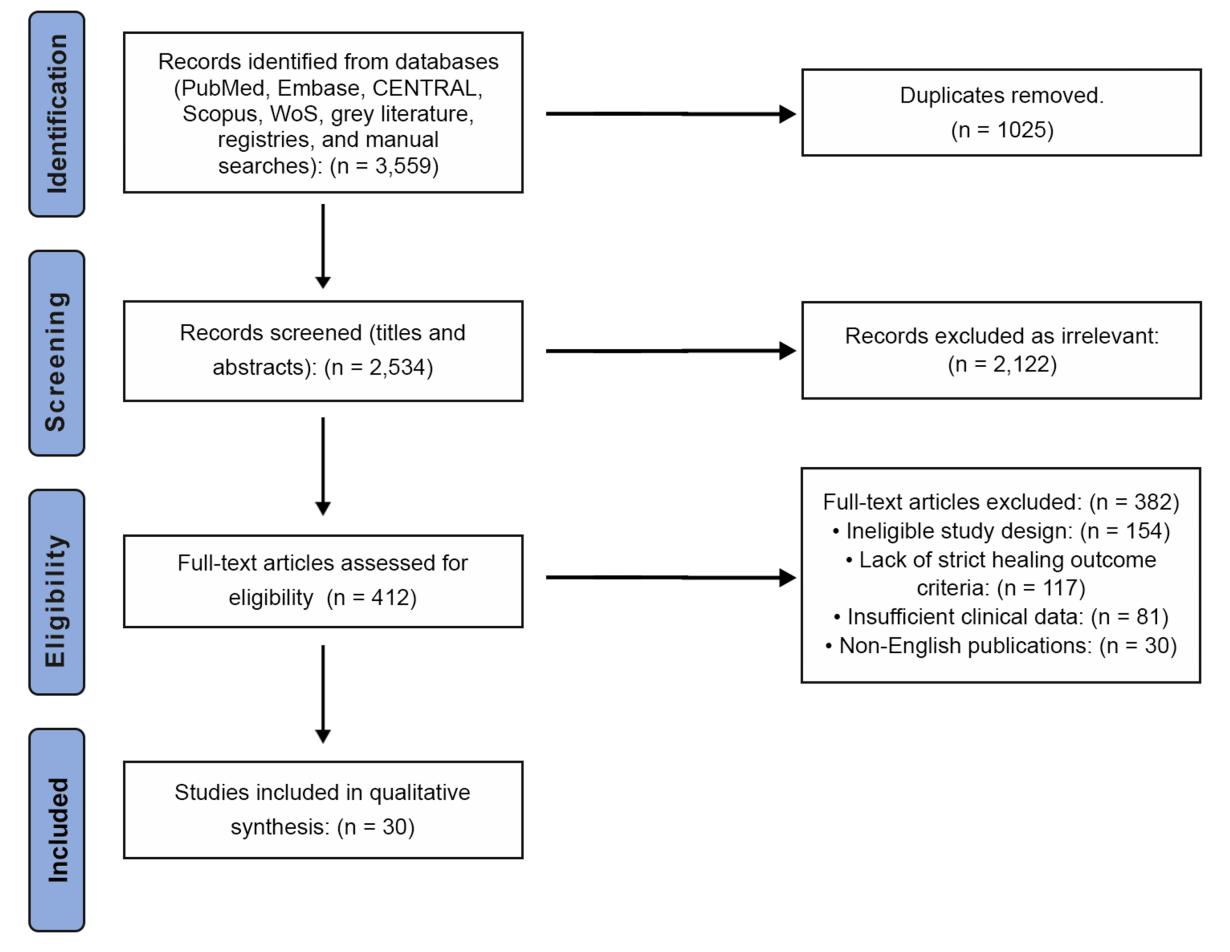
PRISMA 2020 flow diagram of study selection PRISMA: Preferred Reporting Items for Systematic Reviews and Meta-Analyses; WoS: Web of Science; CENTRAL: Cochrane Central Register of Controlled Trials

The study selection process adhered to the PRISMA 2020 guidelines [12]. In total, 3,559 articles were identified. After removing 1,025 duplicate articles, a total of 2,534 unique records remained for screening. Following the screening of titles and abstracts, 2,122 studies were excluded as irrelevant, resulting in 412 articles for full-text assessment. Of these, 382 studies were excluded because they did not meet the eligibility criteria. The main reasons for exclusion were ineligible study design (n=154), lack of strict healing outcome criteria (n=117), insufficient clinical data (n=81), and non-English publications (n=30). Ultimately, 30 records met all inclusion criteria and were included in the qualitative synthesis. Figure 1 illustrates the overall selection process and presents the PRISMA 2020 flow diagram.

*Methodological Characteristics of the Included Studies* The included studies had several variations in design, sample size, follow-up duration, and criteria used to define healing, leading to differences in reported success rates. A detailed summary of their key characteristics is presented in Table 2.

**Table 2:** Methodological characteristics of the included studies on nonsurgical retreatment of teeth with persistent apical periodontitis The sample size is reported as number of teeth unless otherwise specified (roots). RCT: randomized controlled trial; Log. Reg.: logistic regression; ANOVA: analysis of variance; Chi square: chi-squared test; Mann-Whitney U: Mann-Whitney U test; Cox regression: proportional hazards regression; Student's t: Student's t-test; Fisher's exact: Fisher's exact test; Fisher-Freeman-Halton: Fisher-Freeman-Halton exact test

Author	Country	Study design	Sample size (n)	Follow-up	Healing criteria	Reported analysis
Imura et al. (2007) [22]	Brazil	Retrospective cohort	624 teeth	>4 yrs	Clinical + radiographic	Chi square, Fisher's exact, Log. Reg.
Hoskinson et al. (2002) [23]	UK	Ambispective	76 roots	>4 yrs	Clinical + radiographic	Log. Reg.
Gorni and Gagliani (2004) [24]	Italy	Ambispective	452 teeth	2-4 yrs	Clinical + radiographic	Mann-Whitney U
Calışkan (2005) [25]	Turkey	Prospective	86 teeth	2-4 yrs	Clinical + radiographic	Chi square
de Chevigny et al. (2008) [26]	Canada	Prospective cohort	229 teeth	>4 yrs	Clinical + radiographic	Chi square, Fisher's exact, Log. Reg.
Fu et al. (2011) [27]	China	Retrospective cohort	38 teeth	2-4 yrs	Clinical + radiographic	Chi square, Fisher's exact, Log. Reg.
Ng et al. (2011) [28]	UK	Prospective cohort	1314 roots	>4 yrs	Clinical + radiographic	Log. Reg.
Ricucci et al. (2011) [29]	Italy	Prospective cohort	71 teeth	>4 yrs	Clinical + radiographic	Log. Reg.
Krupp et al. (2013) [30]	Germany	Retrospective cohort	48 teeth	2-4 yrs	Clinical + radiographic	Chi square, Fisher's exact, Student's t
Mente et al. (2014) [31]	Germany	Ambispective	30 teeth	2-4 yrs	Clinical + radiographic	Chi square, Log. Reg.
Touboul et al. (2014) [32]	France	Ambispective	108 teeth	2-4 yrs	Clinical + radiographic	Fisher's exact
Azim et al. (2016) [33]	USA	Retrospective cohort	41 roots	2-4 yrs	Clinical + radiographic	Log. Reg.
Nesković et al. (2016) [34]	Serbia	Prospective	49 teeth	2-4 yrs	Clinical + radiographic	Mann-Whitney U
Eyuboglu et al. (2017) [35]	Turkey	Retrospective cohort	110 teeth	2-4 yrs	Clinical + radiographic	Fisher's exact, Fisher-Freeman-Halton, Log. Reg.
He et al. (2017) [36]	USA	Prospective cohort	52 teeth	2-4 yrs	Clinical + radiographic	Fisher's exact
Chybowski et al. (2018) [37]	USA	Retrospective cohort	72 teeth	2-4 yrs	Clinical + radiographic	Chi square
Olcay et al. (2019) [38]	Turkey	Retrospective cohort	101 teeth	2-4 yrs	Clinical + radiographic	Chi square, Fisher's exact, Fisher-Freeman-Halton, Mann-Whitney U, Log. Reg.
Pirani et al. (2019) [39]	Italy	Retrospective cohort	55 teeth	>4 yrs	Clinical + radiographic	Log. Reg.
Goldberg et al. (2020) [40]	Argentina	Retrospective cohort	77 teeth	>4 yrs	Radiographic	Log. Reg.
Mareschi et al. (2020) [41]	Italy	Retrospective cohort	900	>4 yrs	Radiographic	Log. Reg.
Yigit-Özer (2020) [42]	Turkey	Retrospective cohort	83 teeth	>4 yrs	Clinical + radiographic	Holm-Sidak multiple comparative
Stenhagen et al. (2020) [43]	Norway	Retrospective cohort	36 teeth	>4 yrs	Radiographic	Chi square, Mann-Whitney U
Serefoglu et al. (2021) [44]	Turkey	Prospective cohort	103	2-4 yrs	Clinical + radiographic	Log. rank
Signor et al. (2021) [45]	Brazil	Prospective cohort	117 teeth	>4 yrs	Clinical + radiographic	Fisher's exact, Student's t, Log. Reg.
Zhang et al. (2021) [46]	China	Prospective	58 teeth	>4 yrs	Clinical + radiographic	Chi square, Fisher's exact, Log. Reg.
Lee et al. (2022) [47]	USA	Retrospective cohort	165 teeth	>4 yrs	Clinical + radiographic	Chi square, ANOVA
Karaoğlan et al. (2022) [48]	Turkey	Randomized clinical trial	100 teeth	2 yrs	Clinical + radiographic	Chi square, Fisher's exact
Olivieri et al. (2023) [49]	Spain	Longitudinal clinical study	113 (129 teeth)	2-4 yrs	Clinical + radiographic	Chi square, Log. Reg.
Baltieri et al. (2024) [50]	Brazil	Retrospective cohort	80 (120 teeth)	2-4 yrs	Radiographic	Chi square, Fisher's exact
Turan Gökduman et al. (2025) [51]	Turkey	Retrospective cohort	375 (408 teeth)	20 yrs	Clinical + radiographic	Cox regression

Study Design

The reviewed literature (1988-2025) on nonsurgical root canal retreatment outcomes comprises a diverse range of study designs, primarily retrospective cohorts [22,27,30,33,35,37-39,40-43,47,50,51], prospective cohorts [25,26,28,29,34,36,44-46], and ambispective studies [23,24,31,32], along with one randomized clinical trial [48] and a longitudinal clinical study [49]. Overall, these studies demonstrate a methodological trend toward prospective and controlled designs in more recent years, reflecting advances in endodontic techniques, materials, and radiographic assessment tools.

Sample Size

Across 30 studies (1988-2025), sample sizes ranged from 30 to 1,314 teeth/roots, reflecting both small clinical cohorts and large institutional datasets, and this variation contributed to differences in the precision of reported effect estimates, particularly in smaller studies, which was taken into account when judging the certainty of evidence. Smaller cohorts [30,31] provided detailed clinical data, while larger multicenter or institutional studies [28,41] allowed broader outcome generalization. Follow-up periods typically spanned 2-4 years, with several long-term studies exceeding four years [22,28,41,51].

Outcome Assessment and Healing Criteria

Almost all studies applied combined clinical and radiographic criteria for determining healing, as per the guidelines issued by the European Society of Endodontology (ESE) or American Association of Endodontists (AAE). Radiographic healing, involving a reduction or resolution of periapical radiolucency, and absence of symptoms were considered markers of success. However, a few studies [40,41,43,50] that relied on retrospective image-based data collection used only radiographic assessment to determine healing.

Success Rate

Among individual studies, the reported success rates ranged from 61% to 94%, depending on methodology, tooth type, and initial pathology, with the lower end of this range typically observed in earlier cohorts involving teeth with larger preoperative periapical lesions and less favorable baseline conditions. While early studies showed moderate success [24,26], the more recent studies report higher rates due to advanced irrigation and obturation techniques [36,48]. Long-term follow-ups demonstrated stable or slightly reduced success, suggesting that biologic and restorative factors influence late failures [51].

Geographic and Temporal Distribution

Geographic spread: The included studies originate from Europe [23-25,28-32,34,35,38,39,41-44,48,49,51], North America [26,33,36,37,47], South America [22,40,45,50], and Asia [27,46]. Specifically, the majority originated from Turkey, Italy, and the United States, indicating active academic interest in retreatment outcomes in these regions.

Temporal distribution: Studies conducted in the early 2000s were small ambispective and prospective trials (1988-2008). In the 2010s, there was a shift to cohort-based prospective and retrospective designs. More recent studies (2020-2025) included large datasets and multicenter retrospective cohorts with ≥5-year follow-up periods, reflecting methodological maturation and standardization.

Success Rates and Prognostic Factors

Across studies with ≥2-year follow-up and strict criteria, the median success was ~85% (range 61.6-93.1%), with the highest rates under CBCT-based evaluation (93.1%) [46] and conventional radiographic assessment (92.5%) [50]. The lowest rate (61.6%) was associated with extensive periapical lesions [25]. Outcome assessment in nearly all studies relied on combined clinical and radiographic criteria, while a subset also incorporated CBCT-based strict healing evaluation [46,49]. Healing definitions typically followed either strict criteria requiring complete bone fill and an asymptomatic tooth or lenient criteria, where a reduction in lesion size coupled with the absence of symptoms was considered successful.

Treatment outcomes were consistently associated with several prognostic factors. Among preoperative variables, the presence and size of periapical lesions were repeatedly identified as negative predictors [23,25,26,28,29,33,35,38,42,45,48], with several studies using lesion diameter cut-offs of approximately 5 mm (e.g., <5 mm vs. ≥5 mm) to distinguish more favorable from less favorable prognoses [29,38], while vital pulpal status and high quality of the original obturation and coronal restoration were linked to more favorable outcomes [26,29,33,36,42,47]. Intraoperatively, achieving full working length, adequate apical preparation, and avoiding procedural errors or perforations were important determinants of healing [26,28,33,42,44,48,49], whereas canal morphology alterations, missed canals, and overfilling negatively affected success [24,29,33,45]. Postoperative factors, including the quality of the coronal seal and restoration integrity [28,42,47], as well as operator experience and skill [30,31,47], also influenced outcomes. Finally, patient-related variables such as older age [33,35,44] and systemic or immune conditions [33] were occasionally associated with reduced healing potential. Table 3 summarizes the reported success rates and key prognostic factors influencing NS-ReTx outcomes.

**Table 3:** Success rates and factors affecting outcomes in the included studies Reported success rates are based on combined clinical and radiographic criteria unless otherwise stated. NS: not significant; AP: apical periodontitis; PAI: periapical index; S: strict criteria; L: loose criteria; EDTA: ethylenediaminetetraacetic acid

Author	Reported success rate (%)	Prognostic factors reported
Imura et al. (2007) [22]	85.9	Preoperative radiolucency. Patient age. Tooth type
Hoskinson et al. (2002) [23]	78 (Protocol A) and 76 (Protocol B)	Preoperative pulpal status: vital and nonvital. Presence of a periapical lesion-negative prognostic factor. Size of a periapical lesion
Gorni and Gagliani (2004) [24]	69.03	Success is based on the condition of the tooth after initial treatment (86.8% for respected morphology vs. 47% for significant anatomical changes)
Calışkan (2005) [25]	61.6	(1) Previous treatment type. (2) Lesion size
de Chevigny et al. (2008) [26]	82	Quality of the previous root filling. The presence of a root perforation. Preoperative radiolucency. Number of treatment sessions. Previous root filling quality
Fu et al. (2011) [27]	81.8	(1) Inadequate root canal filling. (2) Removal of broken file vs. leaving it (NS). (3) Presence of perforation (NS)
Ng et al. (2011) [28]	80	Absence of a periapical lesion. Smaller lesion size. Absence of a preoperative sinus tract. Absence of inter-appointment flare-up. Achieving patency at the canal terminus. Extension of canal cleaning. Absence of root-filling extrusion. Absence of tooth/root perforation. EDTA solution in retreatment. Abstaining from chlorhexidine. Presence of a satisfactory coronal restoration
Ricucci et al. (2011) [29]	88.6 (tooth) and 90.3 (canals)	(1) Vital pulp cases. (2) Necrotic pulp (no lesion): 89.5% for teeth and 92.3% for root canals. (3) Necrotic pulp (with lesion): 82.7% for teeth and 84.1% for root canals. (4) Lesion size: <5 mm 86.6% success rate and >5 mm 78.2% success rate. (5) Excessive root filling material. (6) Absence of intracanal dressing. (7) Working length. (8) Coronal restoration and post placement (NS)
Krupp et al. (2013) [30]	73.3	(1) Presence of a preoperative lesion. (2) Communication with the oral cavity. (3) Operator experience. (4) Timing of repair. (5) Location and size of the perforation. (6) Material properties
Mente et al. (2014) [31]	86	Provider experience. Post placement
Touboul et al. (2014) [32]	92	Preoperative signs and symptoms. Preoperative root filling density. Preoperative apical periodontitis (AP) status (NS)
Azim et al. (2016) [33]	81.7	(1) Preoperative pulp condition. (2) Procedural errors. (3) Poor root-filling density. (4) Apical extension of filling. (5) Presence of a preoperative periapical lesion (NS). (6) Tooth type or arch (NS). Healing time affected by age, immune conditions, smaller apical preparations, and overextended filling
Nesković et al. (2016) [34]	67.6	(1) Pre-existing periapical status
Eyuboglu et al. (2017) [35]	90.9	(1) Size of preoperative periradicular lesions. (2) Age (NS). (3) Gender (NS). (4) Tooth type (NS). (5) Periodontal defects (NS)
He et al. (2017) [36]	71.2	(1) Quality of previous treatment. (2) Presence of a periapical lesion. (3) Missed or untreated canals. (4) Procedural errors. (5) Quality of coronal restoration. (6) Complexity of root canal anatomy. (7) Patient health
Chybowski et al. (2018) [37]	90.9	(1) Lesion size. (2) Sealer extrusion (NS). (3) Treatment type. (4) Quality of treatment. (5) Preoperative pulp status. (6) Bacterial load
Olcay et al. (2019) [38]	85.1	(1) Lesion size: teeth with lesions <5 mm: 88.6% success rate and teeth with lesions ≥5 mm: 80% success rate. (2) Tooth type. (3) Age and gender (NS). (4) Preoperative factors (other than tooth type) (NS). (5) Intraoperative factors (NS). (6) Postoperative factors (NS). (7) Size of periapical lesion (NS)
Pirani et al. (2019) [39]	87	Maxillary location. Preoperative flare-up. Fracture occurrence
Goldberg et al. (2020) [40]	81.8	(1) Type of treatment (initial vs. retreatment). (2) Tooth location. (3) Overfilling (NS). (4) Extruded material (NS)
Mareschi et al. (2020) [41]	89.8	(1) Preoperative periapical radiolucency
Yigit-Özer (2020) [42]	83	Proper working length. Preoperative periapical lesion. Previous procedural errors. Coronal restoration
Stenhagen et al. (2020) [43]	69.4	(1) Type of treatment performed
Serefoglu et al. (2021) [44]	88	Post-retreatment apical level of the root canal filling. Age (NS). Gender (NS). Soft tissue tenderness (NS). Intraoral swelling (NS). Lesion size (NS). Preoperative PAI score (NS). Pre-retreatment filling level and density(NS). Active exudate drainage (NS). Density of the new filling (NS). Restoration type (NS)
Signor et al. (2021) [45]	80.5	(1) Root curvature. (2) Altered morphology. (3) Apical root resorption. (4) Preoperative lesion size. (5) Apical root resorption. (6) Tooth type. (7) Initial filling quality
Zhang et al. (2021) [46]	93.1	(1) Tooth type. (2) Preoperative lesion size. (3) Quality of retreatment. (4) Coronal restoration
Lee et al. (2022) [47]	86.8	(1) Coronal restoration. (2) Time. (3) Operator experience
Karaoğlan et al. (2022) [48]	88.6 (single visit) and 86.7 (two visit)	(1) Size of the periapical lesion. (2) Preoperative length of the root canal filling. (3) Number of visits (NS)
Olivieri et al. (2023) [49]	S=80.6. L=93	Tooth type. PAI score. Lesion size. Iatrogenic perforation
Baltieri et al. (2024) [50]	92.5	(1) Radiographic restoration of apical transportation. (2) Patient age and gender (NS). (3) Type of tooth (NS). (4) Preoperative characteristics of the tooth (NS)
Turan Gökduman et al. (2025) [51]	85 (after 20 years 60%)	Initial obturation quality. Coronal restoration quality. Preoperative pain. Persistent pain. Periapical lesion size. Tooth type. Operator prognosis rating

Risk-of-Bias Assessment

To evaluate the methodological quality of the included studies, a structured risk-of-bias assessment was performed [19]. The RoB 2 tool was applied to RCTs, while non-randomized cohort and registry-based studies were assessed using domains adapted from the NOS [20]. Key domains considered included randomization or allocation methods, deviations from intended interventions, completeness of outcome data, reliability of outcome measurement, selective reporting, and other potential sources of bias such as confounding, funding, or operator variability. For overall judgments, a conservative "worst-domain" approach was adopted, where the presence of any domain rated as high risk generally resulted in an overall high-risk classification. This dual approach allowed for a consistent and transparent evaluation across heterogeneous study designs. Table 4 presents the risk-of-bias assessments for all included studies.

**Table 4:** Risk-of-bias assessment of the included studies Categories: low risk, some concerns, high risk, and N/A (not applicable to the study design). The RoB 2 tool was applied to randomized clinical trials (covering randomization/allocation, deviations from intended interventions, missing outcome data, outcome measurement, and selective reporting). Observational studies were appraised using NOS-adapted domains mapped to the same constructs. Overall judgment followed a conservative "worst-domain" approach, where a single high risk domain typically yielded an overall high risk. "Radiographic only" indicates outcome assessment based solely on imaging. RoB 2: Cochrane Risk of Bias 2.0; NOS: Newcastle-Ottawa Scale; CBCT: cone-beam computed tomography

Author	Study design	Randomization/allocation (RoB 2)	Deviations from intended interventions	Missing outcome data	Outcome measurement	Selective reporting	Other bias (confounding, funding, etc.)	Overall judgment
Imura et al. (2007) [22]	Retrospective cohort	N/A	Low	Moderate	Low	Low concerns	Moderate (confounding factors)	Moderate
Hoskinson et al. (2002) [23]	Ambispective	N/A	Low to moderate	Moderate	Low	Some concerns	Moderate (selection bias possible)	Moderate
Gorni and Gagliani (2004) [24]	Prospective	N/A	Low	Low to moderate	Low	Some concerns	Moderate (single center)	Moderate
Calışkan (2005) [25]	Ambispective	Not randomized (N/A)	Low to moderate	Moderate	Low	Some concerns	Moderate (confounding, small sample)	Moderate
de Chevigny et al. (2008) [26]	Prospective cohort	N/A	Low	Low	Low	Some concerns	Low to moderate	Low to moderate
Fu et al. (2011) [27]	Retrospective cohort	N/A	Moderate	Moderate	Some concerns	Some concerns	Moderate (small n, selection)	Moderate
Ng et al. (2011) [28]	Prospective cohort	N/A	Low	Low	Low	Some concerns	Low to moderate (large sample helps)	Low to moderate
Ricucci et al. (2011) [29]	Prospective cohort	N/A	Low	Low	Low	Some concerns	Low to moderate	Low to moderate
Krupp et al. (2013) [30]	Retrospective cohort	N/A	Moderate	Moderate	Low (procedure specific)	Some concerns	Moderate (heterogeneous cases)	Moderate
Mente et al. (2014) [31]	Ambispective	N/A	Low to moderate	Moderate	Low	Some concerns	Moderate (small n)	Moderate
Touboul et al. (2014) [32]	Ambispective	N/A	Low to moderate	Moderate	Low	Some concerns	Moderate	Moderate
Azim et al. (2016) [33]	Retrospective cohort	N/A	Moderate	Moderate	Low	Some concerns	Moderate (resident-treated cases)	Moderate
Nesković et al. (2016) [34]	Prospective cohort	N/A	Low	Low	Low	Some concerns	Low to moderate	Low to moderate
Eyuboglu et al. (2017) [35]	Retrospective cohort	N/A	Moderate	Moderate	Low	Some concerns	Moderate (single center, selection)	Moderate
He et al. (2017) [36]	Prospective cohort	N/A	Low	Low	Low	Some concerns	Low to moderate	Low to moderate
Chybowski et al. (2018) [37]	Retrospective cohort	N/A	Moderate	Moderate	Some concerns (single cone + sealer outcome)	Some concerns	Moderate	Moderate
Olcay et al. (2019) [38]	Retrospective cohort	N/A	Moderate	Moderate	Low	Some concerns	Moderate	Moderate
Pirani et al. (2019) [39]	Retrospective cohort (10 yrs)	N/A	Moderate	Moderate to high (long follow-up losses possible)	Low	Some concerns	Moderate (longitudinal attrition)	Moderate to serious
Goldberg et al. (2020) [40]	Retrospective radiographic	N/A	Moderate	Moderate	Some concerns (radiographic only)	Some concerns	Moderate (image only, confounding)	Moderate
Mareschi et al. (2020) [41]	Retrospective cohort (large n)	N/A	Moderate	Moderate	Low (radiographic survival focus)	Some concerns	Low to moderate (single operator but large sample)	Moderate
Yigit-Özer (2020) [42]	Retrospective outcome	N/A	Moderate	Moderate	Low	Some concerns	Moderate	Moderate
Stenhagen et al. (2020) [43]	Retrospective cohort	N/A	Moderate	Moderate	Some concerns (radiographic)	Some concerns	Moderate (small n)	Moderate
Serefoglu et al. (2021) [44]	Prospective cohort	N/A	Low	Low	Low	Some concerns	Low to moderate	Low to moderate
Signor et al. (2021) [45]	Retrospective (data mining)	N/A	Moderate	Moderate	Low	Some concerns	Moderate (modeling/selection)	Moderate
Zhang et al. (2021) [46]	Prospective (CBCT)	N/A	Low	Low	Low (CBCT=more sensitive)	Some concerns	Low to moderate	Low to moderate
Lee et al. (2022) [47]	Retrospective cohort	N/A	Moderate	Moderate	Low	Some concerns	Low to moderate	Moderate
Karaoğlan et al. (2022) [48]	Randomized clinical trial	Low risk (randomized)	Low	Low	Low (clinical + radiographic, blinded assessment unclear)	Some concern (protocol not always reported)	Low to moderate	Some concerns/low to moderate
Olivieri et al. (2023) [49]	Longitudinal clinical study	N/A	Low to moderate	Low to moderate	Low	Some concerns	Low to moderate	Low to moderate
Baltieri et al. (2024) [50]	Retrospective cohort	N/A	Moderate	Moderate	Low (radiographic)	Some concerns	Moderate (single technique)	Moderate
Turan Gökduman et al. (2025) [51]	Retrospective cohort (large, long follow-up)	N/A	Moderate	Moderate to high (long follow-up)	Low (clinical + radiographic)	Some concerns	Serious (confounding, loss to follow-up)	Moderate to serious

**Figure 2:**
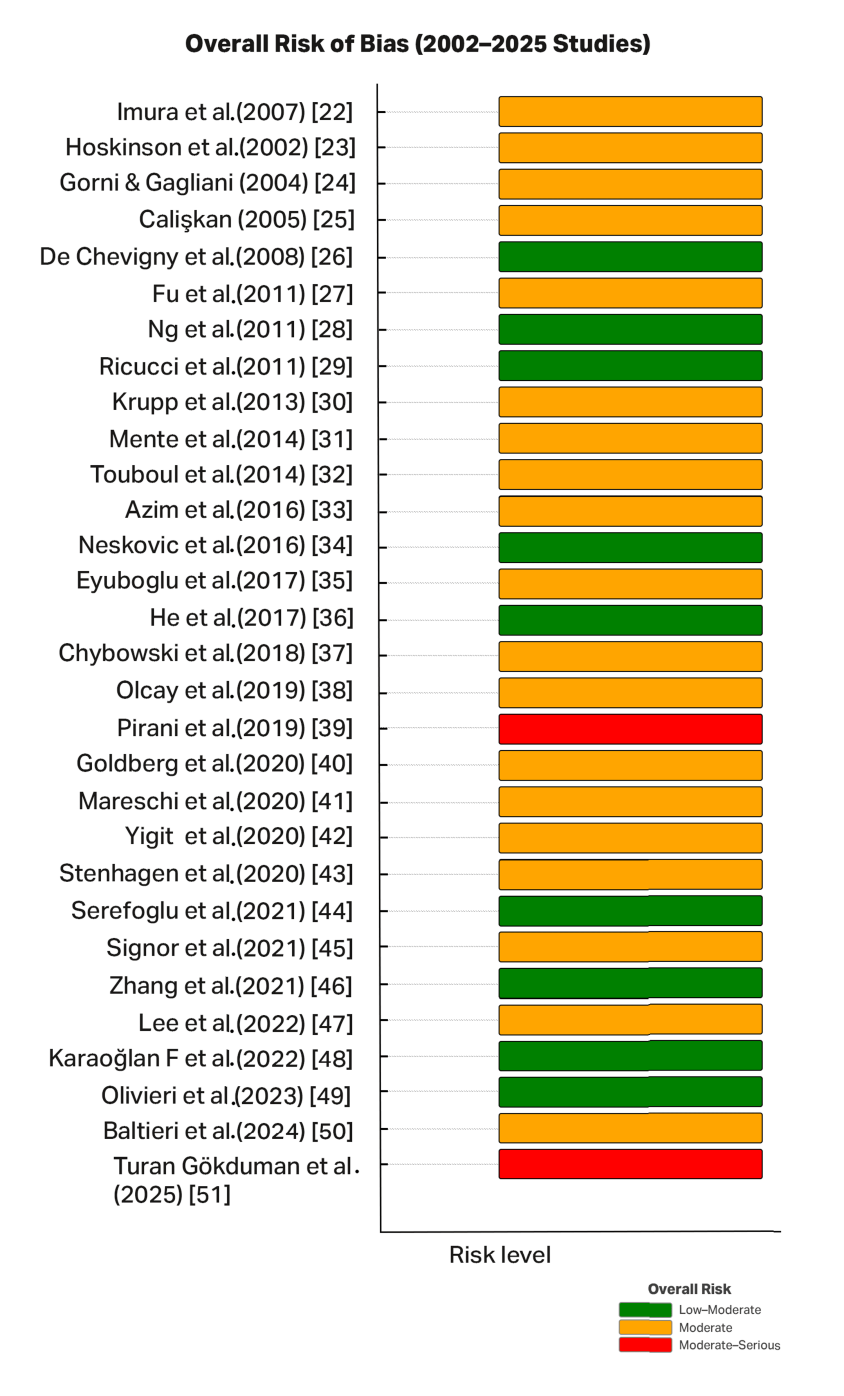
Risk-of-bias traffic light plot for the included studies Risk of bias was evaluated using the RoB 2 tool for randomized trials and NOS-adapted domains for observational studies. Studies were classified into three categories, that is, low to moderate (green), moderate (orange), and moderate to serious (red), based on a conservative worst-domain approach. Most studies were judged at moderate risk primarily because of selection and confounding domains (non-randomized, retrospective designs with limited control of case mix), with additional concerns about attrition in long-term cohorts and, in a few radiographic-only studies, outcome measurement bias; only a small number achieved low to moderate ratings, and two long-term studies showed moderate to serious risk. RoB 2: Cochrane Risk of Bias 2.0; NOS: Newcastle-Ottawa Scale

A bar plot illustrates the overall risk-of-bias judgments for all included studies, showing most as moderate, fewer as low to moderate, and a small subset as moderate to serious (Figure 2).

Interpretation of Risk-of-Bias Findings

The overall risk of bias was assessed for all included studies (n=30) published between 1988 and 2025. Each horizontal bar in the plot corresponds to an individual study, and the color coding reflects the overall judgment of bias risk according to the RoB 2.0 and NOS-adapted domains. In this classification, a low to moderate risk of bias is shown in green [26,28,29,34,36,44,46,49], moderate risk or some concerns in yellow [22-25,27,30-33,35,37,38,40-43,45,47,50], and a moderate to serious risk of bias in red [39,51].

Overall, most studies had a low to moderate risk of bias, indicating generally acceptable methodological quality. A limited number of studies showed serious risks, mainly due to long follow-up attrition or reliance on non-randomized retrospective designs. Notably, significant improvement in study quality was noted in recent studies, particularly prospective and randomized trials published after 2020.

Certainty of Evidence Assessment

To complement the synthesis of clinical outcomes, the certainty of evidence was evaluated using the GRADE approach [21]. GRADE considers five domains: risk of bias, inconsistency, indirectness, imprecision, and publication bias. This framework provides a structured appraisal of the strength and reliability of the evidence regarding NS-ReTx for persistent apical periodontitis.

The summary of findings (Table 5) presents the key outcomes, their estimated effect ranges, and the corresponding certainty of evidence ratings.

**Table 5:** Summary of outcomes and certainty of evidence (GRADE) for nonsurgical retreatment Certainty of evidence was assessed using the GRADE approach, which considers five domains: risk of bias, inconsistency, indirectness, imprecision, and publication bias. Study design was predominantly observational (cohort), downgraded one level; risk of bias was moderate (selection bias, loss to follow-up); consistency showed moderate heterogeneity (success 61-93%); directness was high (direct clinical outcomes); precision was moderate (wide confidence intervals in smaller studies). The overall certainty was judged as low to moderate. High: very confident in the effect estimate; Moderate: moderately confident, with the possibility that the true effect may differ; Low or Very Low: limited confidence GRADE: Grading of Recommendations Assessment, Development, and Evaluation; CBCT: cone-beam computed tomography

Study	GRADE certainty	Justification/interpretation
Imura et al. (2007) [22]	Moderate	Large retrospective cohort, clear inclusion, minor bias due to retrospective design
Hoskinson et al. (2002) [23]	Moderate	Retrospective; confounding and small sample size affected precision
Gorni and Gagliani (2004) [24]	Moderate	Ambispective study; acceptable outcome criteria, moderate selection bias
Calışkan (2005) [25]	Moderate	Prospective design; limited by small sample and single operator bias
de Chevigny et al. (2008) [26]	Moderate	Prospective; robust follow-up but minor inconsistency across operators
Fu et al. (2011) [27]	Moderate	Retrospective; radiographic-based assessment, limited generalizability
Ng et al. (2011) [28]	High	Large prospective study; standardized criteria, strong methodology
Ricucci et al. (2011) [29]	High	Large prospective study; long-term follow-up, consistent methods
Krupp et al. (2013) [30]	Moderate	Retrospective; variable lesion types, moderate imprecision
Mente et al. (2014) [31]	Moderate	Ambispective; good follow-up, limited by selection bias
Touboul et al. (2014) [32]	Moderate	Ambispective; operator variability among postgraduates
Azim et al. (2016) [33]	Moderate	Retrospective; solid design but moderate follow-up loss
Nesković et al. (2016) [34]	Moderate	Prospective; adequate follow-up but small cohort size
Eyuboglu et al. (2017) [35]	Moderate	Retrospective; possible selection bias
He et al. (2017) [36]	Moderate	Prospective; standardized outcome evaluation, moderate sample
Chybowski et al. (2018) [37]	Moderate	Retrospective; sealer type bias possible
Olcay et al. (2019) [38]	Moderate	Retrospective; robust radiographic criteria, limited operator variation
Pirani et al. (2019) [39]	Moderate	Retrospective 10-year data; attrition bias likely
Goldberg et al. (2020) [40]	Moderate	Retrospective; lacks clinical outcome validation
Mareschi et al. (2020) [41]	Moderate	Retrospective; single operator, limited generalizability
Yigit-Özer (2020) [42]	Moderate	Retrospective; fair methodology, moderate precision
Stenhagen et al. (2020) [43]	Moderate	Retrospective; small sample size, moderate indirectness
Serefoglu et al. (2021) [44]	High	Prospective cohort; robust design and standardized evaluation
Signor et al. (2021) [45]	Moderate	Retrospective regression and data mining; selection bias possible
Zhang et al. (2021) [46]	High	Prospective CBCT-based study; strong methodology and high accuracy
Lee et al. (2022) [47]	Moderate	Retrospective; institutional cohort, operator variation present
Karaoğlan et al. (2022) [48]	High	Randomized clinical trial; strong internal validity, low bias risk
Olivieri et al. (2023) [49]	Moderate	Longitudinal; focus on obturation method, moderate indirectness
Baltieri et al. (2024) [50]	Moderate	Retrospective; consistent radiographic criteria, fair sample size
Turan Gökduman et al. (2025) [51]	Moderate	Long-term cohort; some loss to follow-up but robust design

Overall, the included studies demonstrate moderate to high certainty of evidence. High-certainty ratings were observed in well-designed prospective cohorts and randomized clinical trials [28,29,44,46,48]. In contrast, retrospective and ambispective studies were generally graded as moderate certainty, primarily due to potential risks of bias, smaller sample sizes, and moderate imprecision.

Discussion

The present systematic review aimed to consolidate and critically evaluate the available evidence regarding the clinical and radiographic outcomes of NS-ReTx in teeth with persistent apical periodontitis. Across the 30 included studies (randomized trials, prospective cohorts, and retrospective analyses), success rates for strict-criteria healing commonly ranged from the mid-60s to low-80s percent. This supports NS-ReTx as a predictable, conservative approach for persistent apical disease.

Comparison With Previous Literature

Historically, success rates of NS-ReTx have varied widely because of inconsistencies in diagnostic criteria, follow-up duration, imaging modality, and operator technique [22-51]. Across the included studies in this review, the overall success of NS-ReTx is consistently favorable, with reported success/healing rates commonly in the ~70-93% range depending on case mix, follow-up, and outcome definition. The success rates are 78% [23], 69% [24], 61.6% [25], 82% [26], 81.8% [27], ~80% [28], 88.6-90.3% [29], and 86-92% in several cohorts [31,32,41]. However, large specialist series and prospective cohorts [22,28,29] generally report higher success than small retrospective series, a pattern concordant with earlier systematic reviews showing predictable but variable healing after retreatment. More recent technique-specific cohorts (warm gutta-percha, foraminal enlargement + chlorhexidine (CHX)) reported higher success (≥90% in selected series) but are limited by single-technique sampling [49,50]. Overall, findings align with prior reviews that NS-ReTx is an effective conservative option and that outcomes have improved over time with better instrumentation, magnification, and irrigation protocols [28,29,33,46] and are consistent with recent systematic reviews and meta-analyses reporting high pooled success and healing rates for contemporary NS-ReTx [49,52].

Radiographic Versus CBCT-Based Assessment

Several older and contemporary studies used periapical radiographs (2D) plus clinical findings to define healing [23-27,29,32,33,38]. CBCT was used in fewer prospective cohorts [46], and CBCT-based studies tend to report more precise detection of residual lesions and sometimes lower "healed" rates for marginal/borderline cases because CBCT is more sensitive to small periapical changes. Consequently, 2D radiographs may overestimate healing in borderline cases, as they are less sensitive to small volumetric lesions. CBCT offers higher sensitivity, better lesion volume assessment, and earlier detection of incomplete healing, but heterogeneity is increased when CBCT and 2D studies are pooled. From a clinical perspective, routine CBCT for follow-up of all retreatment cases is not currently justified in view of radiation dose and cost; instead, CBCT is best reserved for selected complex, persistent, or surgically considered cases, with periapical radiographs remaining the primary follow-up modality until standardized CBCT-based outcome criteria are widely adopted. This underscores the need to standardize success criteria across imaging modalities, since imaging modality materially affects reported success rates [29,38,46,49]. However, only a small subset of NS-ReTx outcome studies with ≥12-month follow-up used CBCT-based healing criteria. Hence, our synthesis primarily reflects 2D radiographic outcome assessment despite the broader CBCT literature.

Influence of Prognostic Factors

Across the dataset, there is strong, consistent evidence that the outcome of nonsurgical endodontic retreatment depends on the preoperative periapical status and lesion size [23,25,26,28,29,33,35,38,42,45,48,51], quality of previous root filling (density and length) and pre-treatment obturation status [26,28,29,33,36,39], presence of procedural errors (perforation and separated instruments), missed canals, and sealer/extrusion issues [27,29,30,37,49], coronal restoration/seal [28,36,47,51], tooth type and canal anatomy [22,29,38,45], operator experience [31,32,47], and patient factors (age and systemic health) [22,33,35,44]. In short, case selection (lesion size and tooth type), remedial mechanical factors (missed canals and obturation quality), and coronal restoration are the dominant drivers of treatment outcomes, with preoperative periapical status and lesion size emerging as the most consistently significant predictors across studies, as summarized in Table 3.

Heterogeneity and Risk-of-Bias Considerations

Heterogeneity in design is seen, with a mix of retrospective, ambispective, and prospective cohorts and a small number of RCTs. There is also an outcome heterogeneity due to different healing criteria (strict vs. lenient), different imaging modalities (2D vs. CBCT), and variable follow-up thresholds (2-20 years). Some studies had a selection bias (single-center or specialist series). Attrition/missing data in long follow-up studies (e.g., 10-year studies) and lack of blinding of outcome assessment are common. Several retrospective studies relied solely on radiographs without clinical correlation [40,50]. All these factors lead to downgrades in certainty (GRADE) for inconsistency and increase the risk of bias and indirectness in many studies (refer to Table 5). Also, funding sources and conflicts of interest were not consistently disclosed, precluding the assessment of sponsorship bias.

Clinical Implications

Based on the body of evidence, NS-ReTx should be considered a first-line option for teeth with persistent apical periodontitis when retreatment is technically feasible, as success rates are generally high (≈80% or greater in many cohorts) [28,29,33,41,49]. Case selection is critical. Teeth without large periapical lesions, retrievable obturations, and good coronal restorations have better prognoses. For large lesions, complex anatomy, or poor restorative prognosis, alternative options (apicoectomy, extraction, or implant) may be considered, but retreatment often remains reasonable [24,25,29,48]. Use of modern adjuncts (CBCT for diagnosis and complex cases, magnification, NiTi instruments, irrigant activation, and bioceramic sealers) likely contributes to improved outcomes in more recent series [33,37,46,49,50]. A follow-up period of ≥2 years is recommended for the radiographic assessment of true healing, as earlier radiographic "improvement" may not represent complete resolution.

Limitations

There was substantial clinical and methodological heterogeneity due to strict vs. loose healing definitions, mixed imaging modalities (2D radiography vs. CBCT), and disparate follow-up intervals. As these could render pooled estimates misleading, a quantitative meta-analysis was not performed. In addition, effect measures and data formats were not uniformly reported (e.g., proportions without standard errors; unadjusted vs. adjusted estimates), key subgroup data were frequently missing, and some multi-phase cohorts risked double-counting if pooled; under these conditions, a single pooled estimate would be potentially misleading rather than informative. This review was not prospectively registered; to mitigate potential review-level reporting bias, we adhered to the PRISMA 2020 guidelines and prespecified eligibility and data items before screening. However, as the included studies were restricted to English-language publications, this may have introduced language bias and reduced the generalizability of the findings. Most included studies were observational, with potential confounding by operator skill and case complexity. Furthermore, outcome definitions varied across studies from different time periods, and older cohorts relied on non-standardized or analog imaging. Differences in sample size, tooth type, and lesion characteristics are other likely contributors to inter-study variability. Moreover, several recent CBCT-based and bioceramic-focused retreatment outcome studies did not meet our predefined eligibility criteria (e.g., mixed surgical and nonsurgical cohorts, diagnostic-only designs, or follow-up <12 months) and were therefore not included; these emerging areas warrant dedicated future systematic reviews.

Future Research Directions

To strengthen evidence and guide practice, future research should adopt standardized outcome definitions (strict vs. lenient), follow-up intervals, and reporting practices, with the use of CBCT/PAI thresholds where appropriate. Also, there should be consensus on the use of 2D radiography and CBCT, with standardized success criteria bridging the two modalities (potentially including volumetric measures and artificial intelligence (AI)-assisted quantification). In addition, multicenter randomized trials comparing modern retreatment protocols, irrigant activation methods, and bioceramic materials would strengthen causal inference. To further refine prognostic modeling, microbiological/molecular biomarkers can be integrated with time-to-event (survival) analyses. Finally, recent studies have begun to address patient-centered outcomes [36]. Further studies should continue to incorporate patient-centered outcomes (pain, function, and quality of life) and cost-effectiveness alongside radiographic findings when assessing healing criteria.

## Conclusions

NS-ReTx is a dependable, conservative solution for managing persistent apical periodontitis. Contemporary techniques, particularly when applied by experienced clinicians, have strengthened healing prospects and long-term tooth retention. Prognosis is significantly influenced by lesion size, the quality of the previous obturation, the condition of the coronal restoration, and operator expertise. Although methods and follow-up periods vary across studies, the overall finding is consistent: when the tooth is restorable and adequate coronal rehabilitation is feasible, NS-ReTx is a biologically sound and durable option for teeth that have failed initial root canal treatment, whereas extraction or surgical approaches may be more appropriate in cases with poor restorative prognosis, non-restorable root fractures, or inadequate periodontal support.
